# Scalable DB+IR Technology: Processing Probabilistic Datalog with HySpirit

**DOI:** 10.1007/s13222-015-0208-z

**Published:** 2016-01-26

**Authors:** Ingo Frommholz, Thomas Roelleke

**Affiliations:** 1 Institute for Research in Applicable Computing, University of Bedfordshire, Luton, UK; 2 Queen Mary, University of London, London, England

**Keywords:** DB+IR, Probabilistic Datalog, HySpirit, Scalability

## Abstract

Probabilistic Datalog (PDatalog, proposed in 1995) is a probabilistic variant of Datalog and a nice conceptual idea to model Information Retrieval in a logical, rule-based programming paradigm. Making PDatalog work in real-world applications requires more than probabilistic facts and rules, and the semantics associated with the evaluation of the programs. We report in this paper some of the key features of the HySpirit system required to scale the execution of PDatalog programs.

Firstly, there is the requirement to express *probability estimation* in PDatalog. Secondly, fuzzy-like predicates are required to model *vague predicates* (e.g. vague match of attributes such as age or price). Thirdly, to handle large data sets there are scalability issues to be addressed, and therefore, HySpirit provides *probabilistic relational indexes* and *parallel and distributed processing*. The main contribution of this paper is a consolidated view on the methods of the HySpirit system to make PDatalog applicable in real-scale applications that involve a wide range of requirements typical for data (information) management and analysis.

## Introduction

Contemporary retrieval applications have to handle large amounts of structured and unstructured data in various ways to provide the user with useful means to satisfy complex information needs. For instance, a system is supposed to give the user the option to perform a quick simple search as well as an advanced search. Users may want the system to perform a deep analysis during retrieval time to increase the quality of the result set, for instance by flexibly incorporating external knowledge sources like an ontology or synonyms for query expansion. Search and retrieval may be performed combining different kinds of structured data (e.g. attribute-value pairs like ‘name’, ‘location’, ‘year’ with potential textual or numeric content) with semi-structured or unstructured data (e.g. a ‘description’ field which contains textual data).

To support the creation of flexible information search services that are able to handle complex information needs with a different level of complexity, we need frameworks that are able to handle structured and unstructured heterogeneous content. These frameworks should offer knowledge engineers the expressiveness to describe, implement and flexibly adapt complex retrieval functions within a short time to application. Providing different search strategies involving heterogeneous, structured and unstructured data, possibly distributed across different network nodes is a tedious task that requires sophisticated, efficient, effective and expressive solutions. Existing enterprise search solutions like Lucene^1^ and its derivates like Solr, Elasticsearch and Nutch as well as research prototypes like Terrier [11] or Indri^2^ are tailored and optimised to efficiently perform mainly fulltext search tasks. The search functionality is usually embedded in the source code written in the underlying imperative or object-oriented programming language. However, similar to the separation of data and code that motivated the development of database management systems, we argue that the implementation of search strategies should be as independent as possible from the remaining code [5]. This way, new search strategies and functionality can be provided and optimised as well as new distributed data sources can be integrated without the larger effort of reimplementing existing code, which also involves deep programming knowledge not always available to domain experts. This finally leads us, given a sufficient and effectively implemented level of abstraction, to the separation of the “what” from the “how” [2]. To this end, probabilistic Datalog (*PDatalog*) has been proposed as a description-oriented logic language combining information retrieval and database features [4]. As a probabilistic extension of Datalog and based on a probabilistic relational algebra (PRA), PDatalog offers structured, ‘database-like’ elements (such as relations and tuples) and combines them with concepts known from information retrieval to handle uncertainty and vagueness.

In this paper, we present a PDatalog implementation called HySpirit that implements probabilistic logics and offers specific probabilistic operators. The expressiveness of the implemented PDatalog layer allows knowledge engineers to swiftly describe complex retrieval strategies, which can be executed efficiently. HySpirit was first discussed in [8], but with a scope on introducing four-valued probabilistic Datalog, a variant of PDatalog that can cope with additional truth values and an open world assumption. In this paper, we will focus on PDatalog and on some salient aspects of HySpirit that have been in the centre of our work in the recent years: a relational Bayes operator for efficient probabilistic inferencing; vague/fuzzy predicates for strict and vague reasoning; a probabilistic relational index for fast access; distributed parallel processing. These aspects will be discussed in the next section. Afterwards we will present some application examples in Section 3.

## Processing Probabilistic Datalog – The HySpirit System

In this section we present some of the main features of the probabilistic Datalog layer as implemented in HySpirit. We start with a general description of PDatalog before we discuss in more detail some salient features of HySpirit PDatalog.

### PDatalog: Syntax and Semantics

**Fig. 1:**
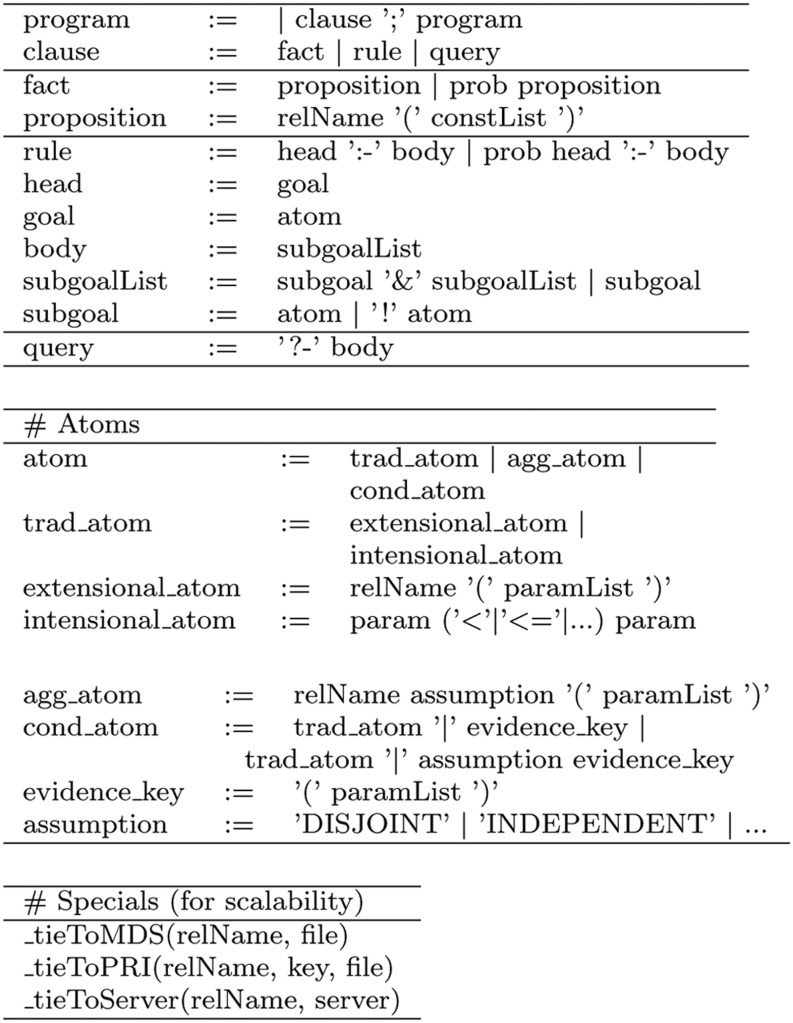
HySpirit PDatalog Syntax: Main Symbols. PDatalog is an extension of Datalog, and the extension is regarding probabilities in facts and rules. Special to HySpirit PDatalog are (1) aggregation and conditional atoms (to describe frequency-based and information-theoretic probability estimations), and (2) a variety of special relations (names start with and the engine interprets those special facts).

Figure 1 shows an extract of the syntax of PDatalog.

A program is a sequence of clauses. A clause is a fact, a rule or a query. A fact assigns the probability of “true” to a proposition. If no probability is given, then *P*(proposition is true) = 1. In four-valued Datalog [8], the truth value “false” can be specified by using “NOT proposition”, but for the purpose of this paper we do not extend on this facility.

A rule comprises a probability (optional), a head and a body. A head is a goal, and a body is a list of subgoals. A goal is an atom, and a subgoal is an atom or a negated atom. In four-valued Datalog, the goal (head) can be of the form “NOT atom”.

For probabilistic facts, the probability reflects the probability of the truth value given the proposition:

*P*(proposition is true). For probabilistic rules, the rule probability is interpreted as a conditional probability:

*P*(body is true). Specific to HySpirit PDatalog is the expansion of an atom into various types of atoms. In this paper, we consider the main three types:

1. traditional atoms: relName(paramList); for example, “isaSailor(Person)” is an atom.
2. aggregation atoms: relName assumption(paramList); for example, “retrieve SUM(Document,Query)” is an aggregation atom typically used in a rule head. Aggregation atoms were introduced in 2002, to provide an extensional way to specify the assumption; the intensional approach (as proposed in [7]) is less scalable than the extensional variant.
  The main assumptions are: DISJOINT, INDEPENDENT and SUBSUMED. They correspond to the usual assumptions applied in probability theory. SUM is a synonym for DISJOINT, and MAX is a synonym of subsumed. The HySpirit engine interprets the assumptions to decide about the aggregation of tuple probabilities.
3. conditional atoms: for example, “nationality_job(Nation, Job)$|$(Job)” is a conditional atom, where “(Job)” is the evidence key. Essentially, the engine divides each tuple probability by the evidence probability. This leads to the conditional probability *P*(Nation|Job).
  Conditional atoms support the usual assumptions (DISJOINT, INDEPENDENT and SUBSUMED), and in addition, there are information-theoretic assumptions such as MAX_InvValueFreq and SUM_InvValueFreq. For the case of IR, the synonyms are MAX_IDF and SUM_IDF. These assumptions allow for the specification of idf-based probabilities [12].
  Note that for conditional atoms, if no assumption is specified, then DISJOINT (sum of the probabilities of the evidence tuples) is the default.

The sections to follow focus on the particular aspects that were added to HySpirit PDatalog in the period 2002 to 2010.

Section 2.2: Conditional Atoms: The Relational Bayes
Section 2.3: Vague (Fuzzy) Predicates
Section 2.4: Probabilistic Relational Indexes (PRIs)
Section 2.5: Parallel and Distributed Processing

### Conditional Atoms: The Relational Bayes

One of the main short-comings of the 1st-generation PDatalog (1995-2000) is the need to explicitly specify probabilities (rule or fact probabilities). Required are facilities that allow for describing probability estimation. The following program show-cases *conditional atoms*, which are a main concept in HySpirit PDatalog.

**Fig. 2:**
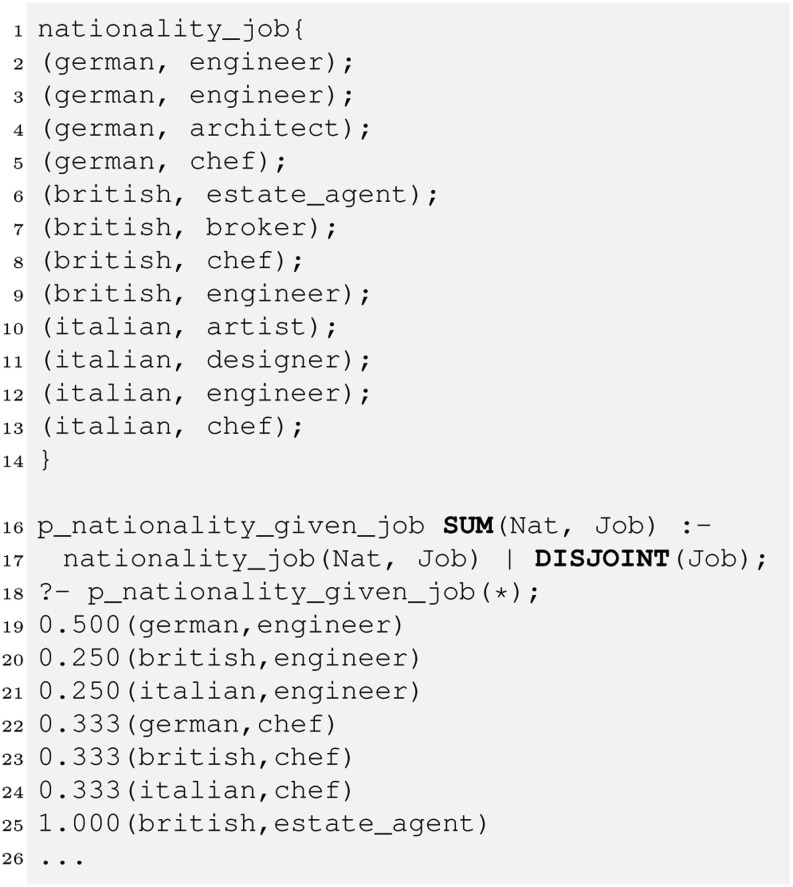
Conditional Atoms (Relational Bayes). Example: For a deterministic relation “nationality_job(Nation,Job)” the rule for “p_nationality_given_job” computes the conditional probability $P({\rm Nation}|{\rm Job})$. The conditional atom consists of the traditional part and the *evidence key*, “(Job)”.

While [16,13] describes the *relational Bayes* and the translation of probabilistic SQL (ProbSQL) to probabilistic relational algebra (PRA) expressions, we provide here for the first time the semantics for PDatalog.

The translation of a PDatalog (PD) conditional atom to the respective PRA expression is indicated by the following mappings between PD and PRA:

PD: relName(paramList1) $|$ assumption (paramList2)

≡

PRA: Project[paramList1](

Bayes assumption[paramList2](relName))

To illustrate, consider an example where for a set of tuples with nationality and job, we wish to compute the probability $P(\textrm{nation}|\textrm{job})$.

PD: nationality_job(Nation,Job) $|$ DISJOINT (Job)

≡

PRA: Project[$Nation,$Job](

Bayes DISJOINT[$Job](nationality_job))

In addition to the usual assumptions already introduced (DISJOINT, INDEPENDENT, SUBSUMED), there are geometric (EUCLIDEAN), logarithmic (MAX_LOG) and information-theoretic (MAX_InvValueFreq, MAX_IDF) assumptions to provide high-level facilities to implement retrieval models.

### Vague (Fuzzy) Predicates

Another specific feature of HySpirit is the integration of vague or fuzzy predicates [4] into PDatalog.

**Fig. 3:**
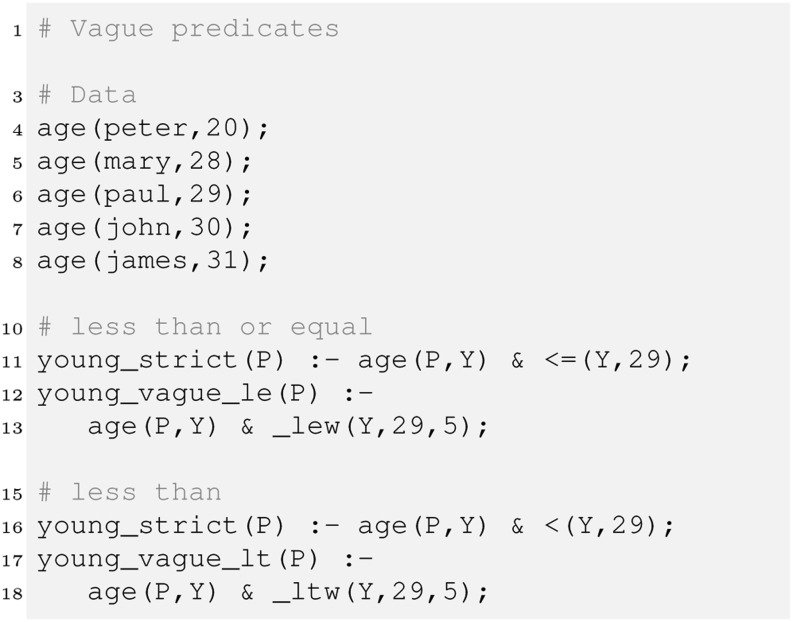
Vague predicates: Example: Reasoning over age with strict and vague predicates.

This is relevant for the retrieval of any object, let it be cars, computers or persons. There is always the problem that we over-specify the query. For example, we want cars with mileage less than 60,000 and price less than 10,000, and the retrieval engine fails to deliver that great value car with 61,000 miles. Similar for recruitment, where human resources want to employ someone young, but the candidate a bit older could be relevant as well. Figure 3 illustrates the usage of vague predicates (in HySpirit PDatalog, these are special atoms starting with “_”).

The program demonstrates the specification of rules for strict and vague reasoning over the age of persons. For the relation age, Peter, Paul and Mary would be considered young. John and James will not be retrieved by the strict rules. The vague rules will include them.

Vague predicates can be parametrised with a third parameter, the “width”. The example applies the width 5, which means for _lew (less-equal-width) that ages $<= 29+5/2$ will be considered with decreasing probability. For _ltw (less-than-width), probabilities are generated for the interval $[29-5/2; 29+5/2]$. The effect of the width is illustrated in Fig. .

**Fig. 4:**
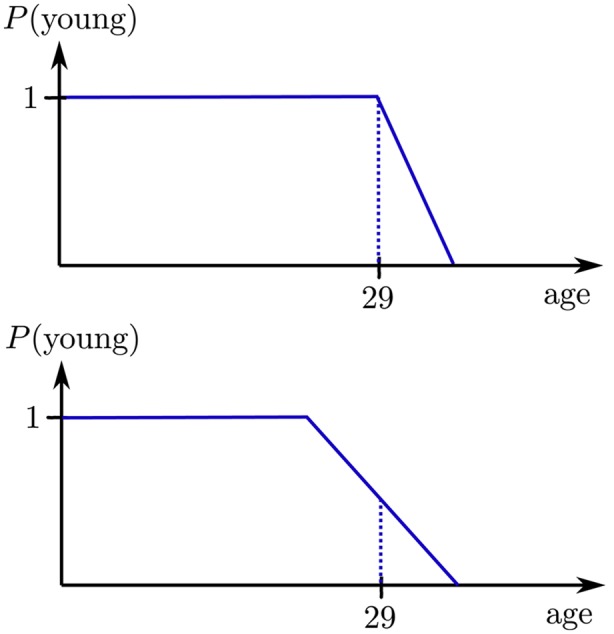
Vague predicates: less equal (top) and less than (bottom): for _lew (less equal, given width), the probability of the value to compare with is $P(29<=29)=1.0$; for values in between 29 to $29+\textrm{width}/2$, the probability is in $(1.0;0.0)$; for _ltw (less than, given width), the probability of the value to compare with is $P(29<29)=0.5$ (note ‘$<$’ is interpreted in a fuzzy way here; for the classical strict interpretation, $P(29<29)$ would of course be 0); for values in between $29-\textrm{width}/2$ to $29+\textrm{width}/2$, the probability is in $(1.0;0.0)$

For less-equal, persons with age up to 31 will still be retrieved. $P(30<=29)=1-2\cdot(30-29)/5 = 3/5$, and so forth. For 32, the vague predicate function returns $1-2\cdot(33-29)/5$, and the value is less than 0, since 33 is outside of the window. Therefore $P(32<=29)=0$, and zero probability tuples will be discarded.

The width controls the slope. The slope is the same for less-equal and less-than, just the interval is different. Similar procedures apply for greater-equal and less-than. The vague equal is simply a triangle over the value to compare with. It is recommended to define attribute-specific rules for comparing values as shown for age, since the requirement for the slope (the width) typically differ. For example, width = 5 for age, whereas width = 2,000 for car prices, and width = 10,000 for mileage. There are various parameters to customise fuzzy predicates.

### Probabilistic Relational Indexes (PRIs)

**Fig. 5:**
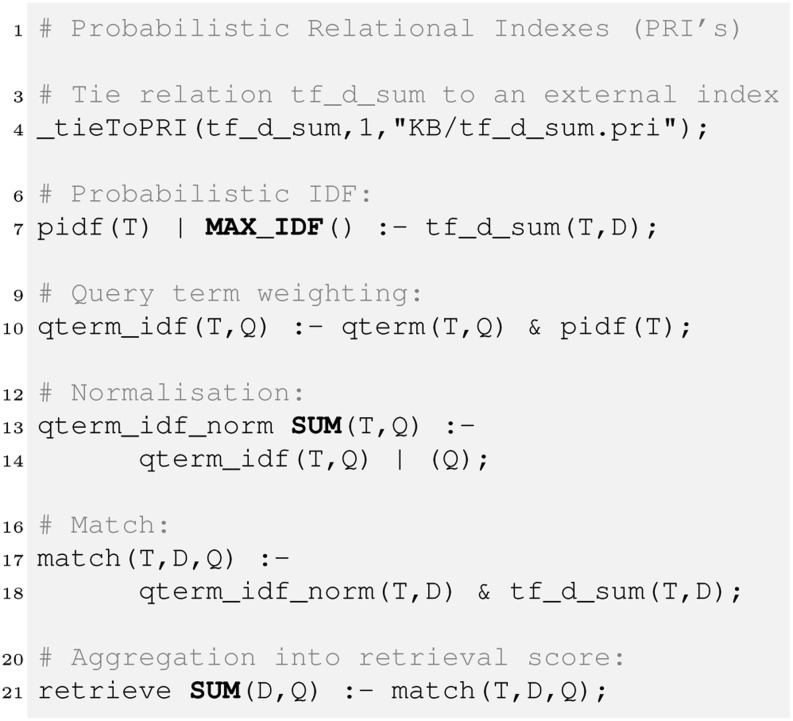
Probabilistic Relational Index (PRI): Example: Tie the first attribute of a relation, here tf_d_sum[$1], to a persistent index.

To store and efficiently access large amounts of data, scalable indexing mechanisms are required. In HySpirit, this is provided by so-called *probabilistic relational indexes (PRI)*. Figure 5 shows how to tie the attributes of a relation to a persistent index.

The relation tf_d_sum(Term,Doc) is a probabilistic relation reflecting a TF quantification. The special _tie(RelName,AttributeSpec,PRI_File) ties the specified attributes to the persistent index. The tied relation is used in the rules for pidf and match. The usage of PRIs means that the fetch of tuples in pidf has complexity $O(1)$ since the PRI delivers the respective term probability. The join of qterm_idf_norm and tf_d_sum is efficient since the (term,doc) pairs are retrieved via the index over the terms (first column in tf_d_sum).

#### Distributed PRIs

**Fig. 6:**
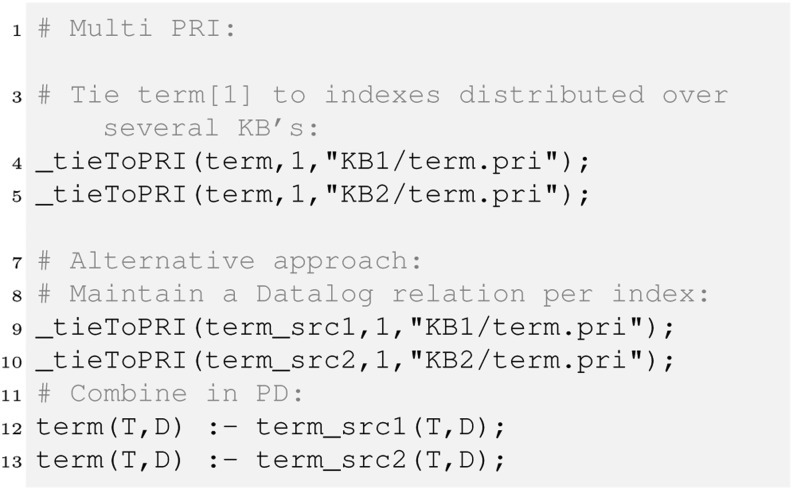
Multi-PRI: Example: The relation “term” is tied to several PRI files. Alternatively, one can use a relation name per index, and merge in the PD program.

For scaling the application, HySpirit provides a mechanism referred to as “multi-pri”, which is a facility to distribute PRIs. Figure 6 illustrates that a relation key can be tied to several PRIs. The example shown ties the index for “term[1]” (first column of the term relation) to two PRI files in different knowledge bases.

This facility allows for dealing with hundreds of millions of tuples. The distribution of tuples over several PRI files is recommended for various reasons. For example, most Unix tools can be applied very efficiently to streams of ten up to one hundred million tuples (corresponds to approximately 50 – 500 MB files, for an average tuple length of 50 characters). Obviously, compression could be used, but this does not change the overall argument, it just increases the data volume sensible per file.

There are numerous challenges regarding multi-PRIs, as for some algebra expressions (e.g. distinct projections or Bayes operations) the aggregation over index elements is required. [9] provides some details about methods for index selection that are useful once a key is to be tied to more than ten indexes. Overall, the facility of multi-PRIs allows for probabilistic reasoning over millions of tuples. For a 4-core 8-GB machine, this distributed indexing strategy scales to 100 million tuples per relation.

### Parallel and Distributed Processing

**Fig. 7:**
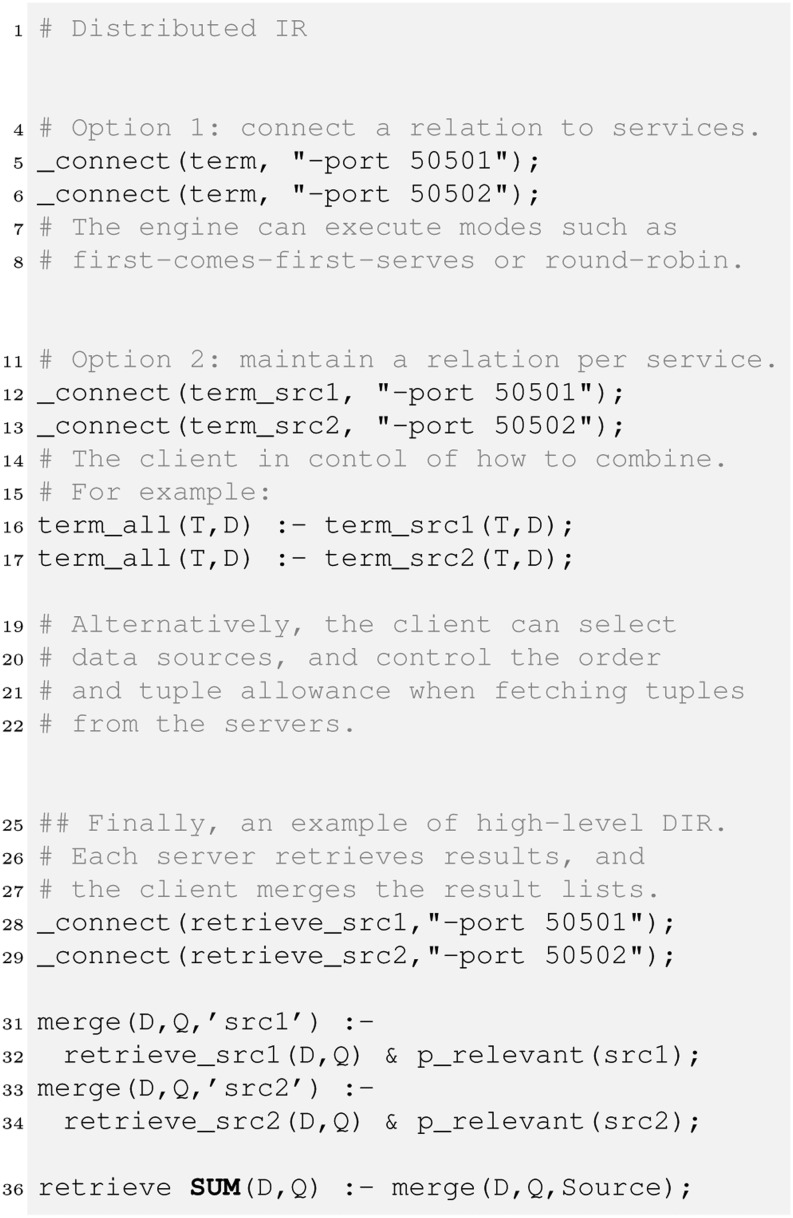
Distributed Processing: Example: The PD engine executing the above program connects to various servers for fetching tuples of the connected relations. The facility can be used to model probabilistic source selection.

The next level of scalability is distributed processing. A client engine connects to multiple server engines that serve probabilistic relations. Figure 7 illustrates a set-up where the relation “term” is connected to HySpirit servers listening on the respective ports.

The client engine reads the PDatalog file with the special _connect directives. Then, if a query involves access to connected relations, the clients sends a request (usually in PRA or PDatalog language, but eventually also in SQL or ProbSQL [16]) to the server engines, and the server returns the respective tuples. [9] provides more details about the client-server architecture to scale applications.

## Application Examples

Having introduced PDatalog and some of its features enabling effective and efficient processing of large volumes of data, we provide some examples of its potential application. We will first look at a full implementation of a full-text search engine. Afterwards we will discuss a more complex example that integrates factual (DB) knowledge with textual (IR) knowledge to implement different retrieval strategies.

### Full-text Search

A potential application of the Bayes operator is a probabilistic variant of the famous TF-IDF ranking formula widely used in information retrieval. Here we are interested in estimating the within-document term frequency ${\rm tf}(t,d)$ of a term *t* in a document *d*. There are several variants of computing this value [14]. One way is using the probability $P(t|d)$ as

1 $${\rm tf}(t,d) = P(t|d) = \frac{occ(t,d)}{\sum_{t'\in d}occ(t',d)}$$

with $occ(t,d)$ being the number of occurrences (or locations) of term *t* in a document *d*. In a similar way we could also compute the probability $P(t|q)$ for a query *q*. The inverse document frequency ${\rm idf}(t)$ of a term *t* is calculated as the negative logarithm of the number of documents a term appears in. In a probabilistic scenario, a normalised ${\rm idf}$ value like

2 $$P_{\rm idf}(t) = \frac{{\rm idf}(t)}{\rm maxidf}$$

can be used (with ${\rm maxidf}$ as the maximal ${\rm idf}(t)$ over all terms). The score for each document *d* w.r.t. a query *q* is then computed as

3 $$score(d,q) = \sum_t P(t|q) \cdot P(t|d) \cdot P_{\rm idf}(t).$$

**Fig. 8:**
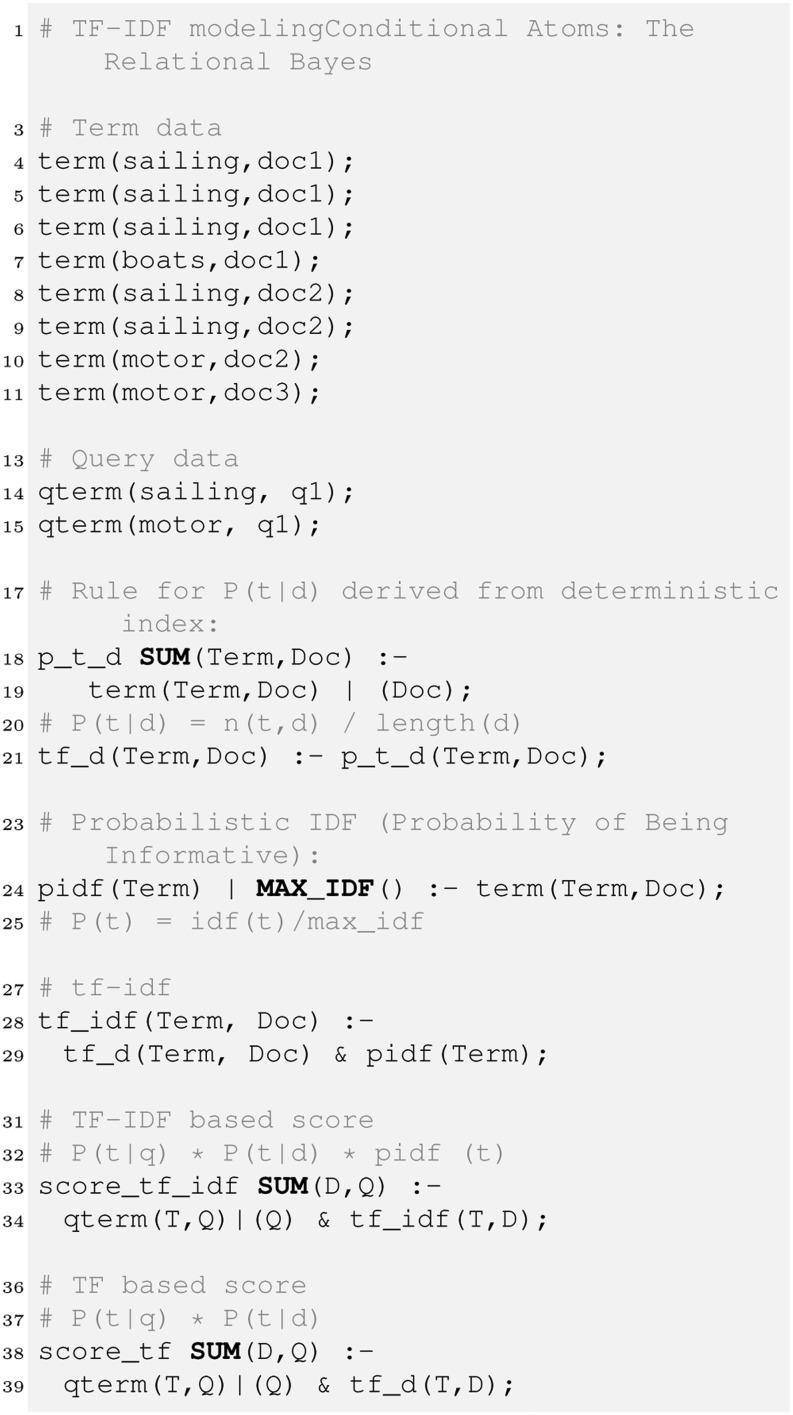
TF-IDF-based full-text search using Relational Bayes utilising frequency-based within-document probability $P(t|d)$ and max_idf.

Figure 8 shows an example PDatalog program to create a TF-IDF-based ranking of documents using the Bayes operator. Lines 4–11 show a toy database that defines the term relation. Here we record each occurrence of a term in a specific document, which may be the result of some tokenisation process – the term ‘sailing’ appears 3 times in document doc1, 2 times in document doc2, etc. Lines 14–15 show the relation qterm that show an example query (labelled q1) for ‘sailing motor’. Line 18 shows how the Bayes operator is applied to compute the probability $P(t|d)$. Here, the 2nd column of the term relation is the evidence key. Tuples in the term relation are grouped by the evidence (in this case documents). The SUM keyword instructs the PDatalog engine to compute $P(t|d)$ based on the sum of the probabilities for each evidence key, thus implementing Eqn. 1 along with line 21. Line 24 shows the implementation of Eqn. 2 using the MAX_IDF operator. Alternatively, the SUM_IDF operator offered by HySpirit would compute Eqn. 2 based on the sum of all idf values and not the maximum one. Line 28 combines tf and idf values. The evaluation of the join operator ‘&’ takes places according to the rules of probability theory. Given that the involved events are independent, the join computes the product of the single probabilities, i.e. $P(t|d) \cdot P_{idf}(t)$ in this example, and assigns the resulting joint probability to the tuple resulting from the corresponding valuation. Finally, the rule in line 33 combines all evidence to compute the score as in Eqn. 3. Note the integrated Bayes operator in the ‘qterm(T,D)$|$(Q)’ conditional atom in line 34 and 39, respectively.

Line 38 contains a rule that provides an alternative implementation of the scoring function, which is only based on tf. Applications could instantly decide which retrieval function is the most suitable one, for instance if the more costly idf computation may not be feasible (for instance in real-time streams

like Twitter).

Besides TF-IDF, many other prominent retrieval functions (like BM25 and Language Models) can be expressed in a similar fashion [15].

### Complex Factual and Textual Search

We turn to a more complex example now to explain some of the advanced DB+IR concepts in HySpirit. The scenario is a search engine for used cars. Consider the following toy database:

**Figure:**
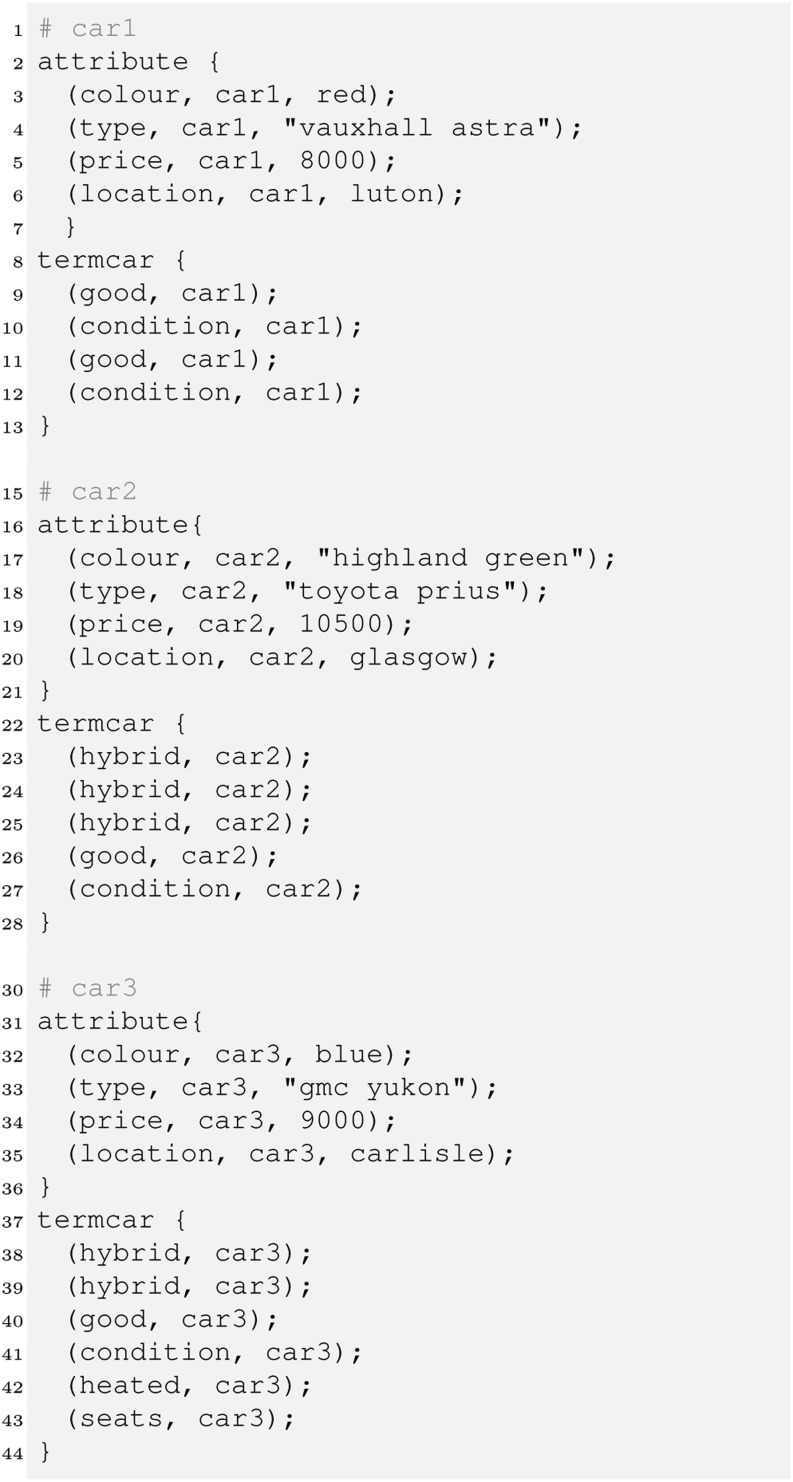

There are three cars with IDs car1, car2 and car3. The system offers different attributes (like colour, type, price, location), which are stored in the attribute relation and also a full-text description of the respective car, with extracted terms and their context (car descriptions) in the termcar relation. For instance, the term ‘hybrid’ appears 3 times in the car2 description, twice in the car3 description, etc. This implements the idea of a generic object-relational content model as proposed in [1].

Note that we provide the toy database directly here; in a productive environment, the termcar and attribute relations would be in one or several PRI’s and, in case for instance of distributed IR on a cluster, connected via the _connect statement as discussed in the previous section. For dealing with a potentially very large relation termcar, rules involving the relation will be materialised, and the rule head will be indexed. For example, the tf_sum rule (see the program below, line 60) will be evaluated by HySpirit taking into account existing indexes in the knowledge bases.

Let us consider two queries, one asking for all cars offered in Scotland, one looking for a Toyota Prius in a good condition for up to £ 10,000.

**Figure:**
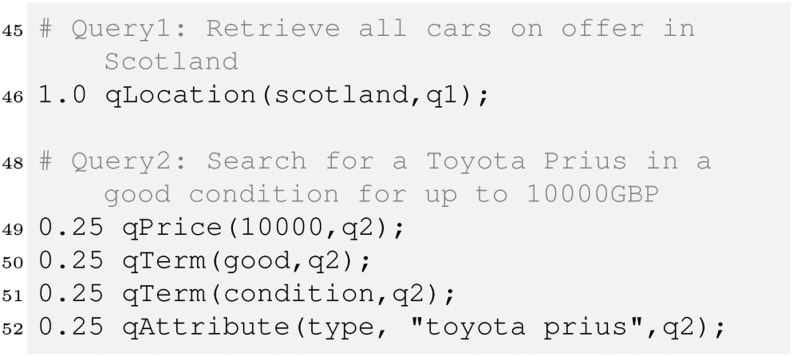

Query 2 (q2) is a simple example of querying factual knowledge (attribute and price) and (textual) content [1]. The single query facts are normalised so that their weight sums to 1. Instead of a uniform value 0.25 we may have provided different values, for instance to emphasise the importance of some of the query aspects. Another option would be to compute these weights from raw data using the Bayes operator in a similar way as described in Section 2.2.

In our example we want to give price and location attributes an extra treatment, so we apply a technique called *semantic lifting* [1] to create corresponding rules. Furthermore we compute the tf as in the previous section.

**Figure:**
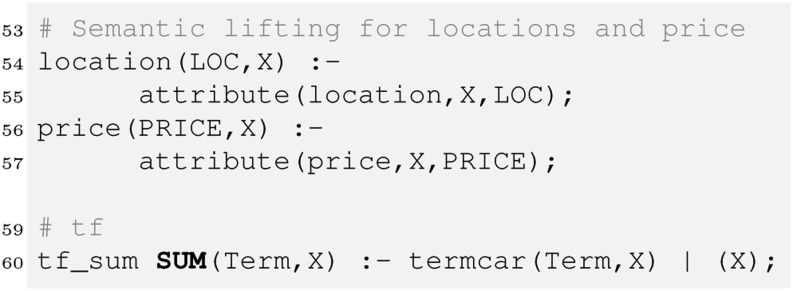

We now create some rules that allow us to collect the matching evidence which can be useful for instance to explain to the user why an item was retrieved. In our example, there are 4 different kinds of such evidence – for locations, prices, text and any other attribute. This can be expressed as follows.

**Figure:**
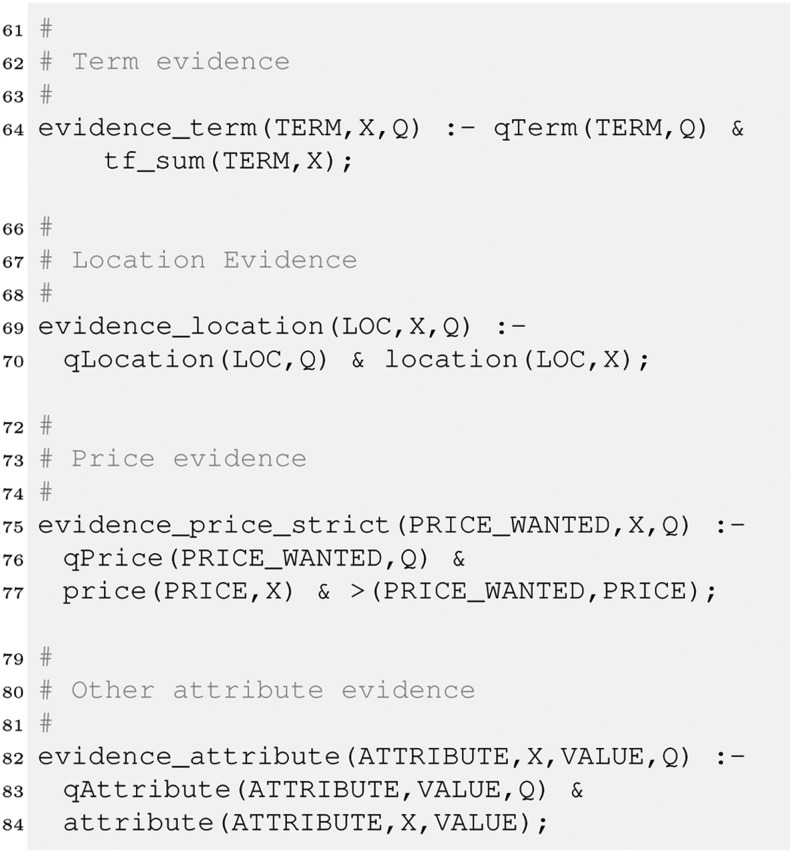

To compute the price match we use a strict operator, which means only items below the price indicated by the user will match. Finally, we have to combine the evidence. We will see later there are several ways to do so, each of them may be implemented as its own retrieval strategy. We therefore introduce a rule defining retrieval_strategy1 along with the retrieve relation that sums the evidence to compute the final score.

**Figure:**
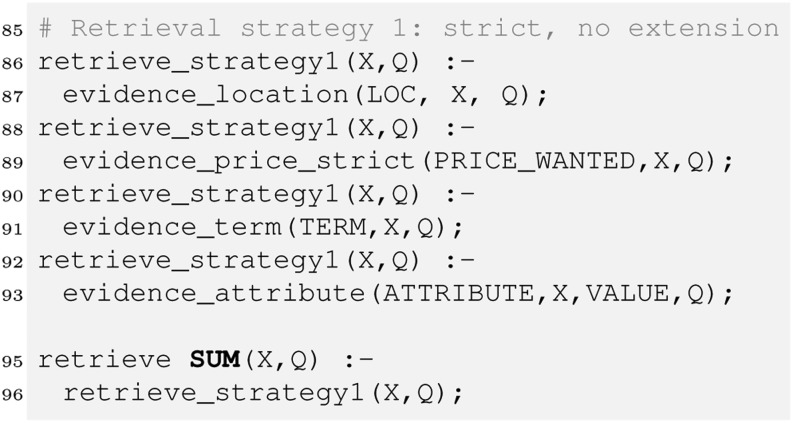

Aggregating the evidence this way implements a *best match* strategy in contrast to the *exact match* strategy prominent in typical databases. Best match means all items (cars here) that match the whole query would be ranked ahead of those matching the query only partially. In exact match, only items fully satisfying the query would be retrieved. A query would return the following ranked list of all Toyota Prius in a good condition for up to £ 10,000. In a similar way we can now query the evidence relations (line 64–82). This would reveal that car1 and car3 matched mainly due to the textual description as well as the price, whereas car2 was retrieved mainly because it is a Toyota Prius.

However, we may argue car2 should be first in the ranking as it is only a near miss regarding price. We can fix this by allowing for vague predicates as discussed in the previous section:

**Figure:**
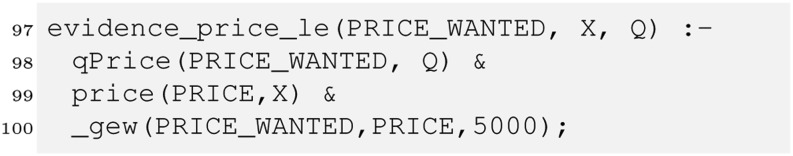

Furthermore, if we look at our query q1 about cars on offer in Scotland, this query would not retrieve anything in the current setting, although car3 with location Glasgow should be retrieved. We therefore introduce a simple location ontology and integrate it into our retrieval strategy.

**Figure:**
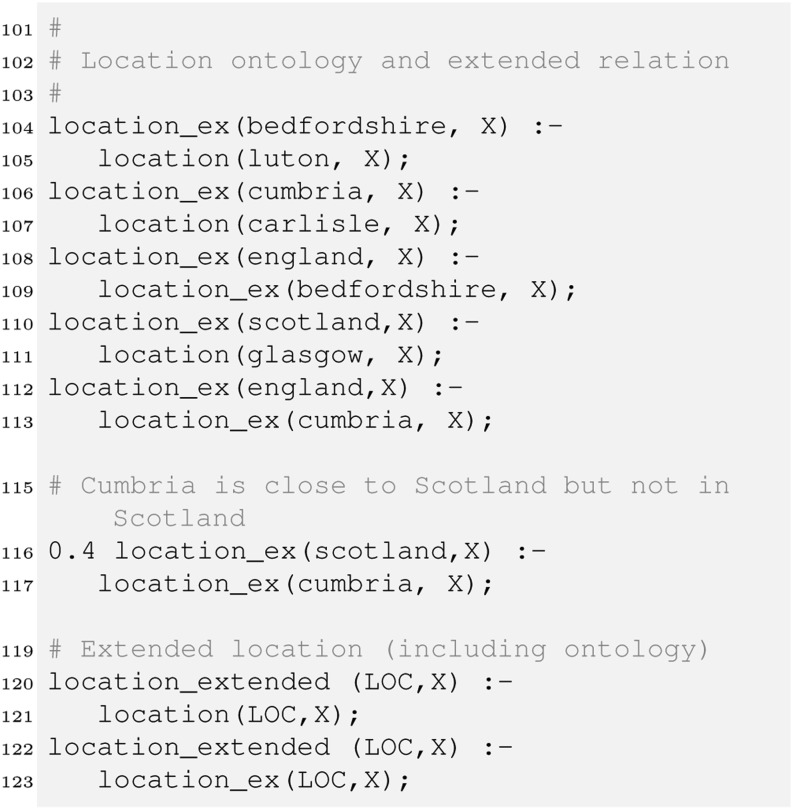

The definitions of extended locations in location_ex state that every item in Luton is in also one in Bedfordshire, every item (car) in Glasgow is also in Scotland, etc. Line 116 is interesting in that respect. Here we state that a customer interested in cars in Scotland might to a lesser degree (hence the 0.4^3^) also be interested in cars in Cumbria, which shares a border with Scotland. The location_extended relation combines the location and the derived location_ext attributes.

Finally, we define our second retrieval strategy which utilises our location ontology as well as the vague price comparison:

**Figure:**
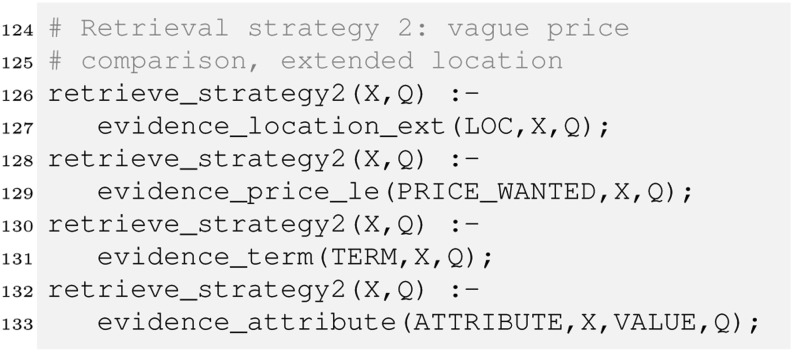

We modify the retrieve rule in line 93 to make retrieval strategy 2 our default strategy.

**Figure:**
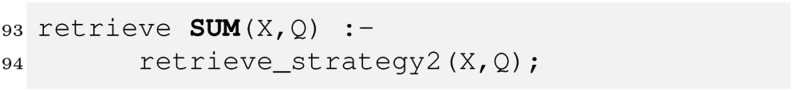

Now, processing the first query (cars on offer in Scotland) would yield the following result:

**Figure:**
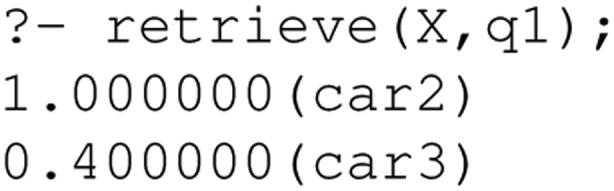

car2 is retrieved as it is located in Glasgow and the system infers this is in Scotland. car3 is also retrieved (to a lesser degree) as it is located in Carlisle, Cumbria, not too far from the Scottish border. We can also see how the ranking for the second query changes due to the vague predicate:

**Figure:**
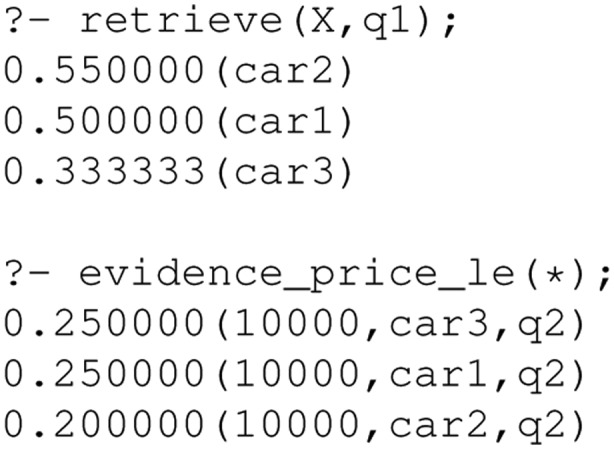

The price evidence shows us that car2 now matches the price query, albeit to a lesser degree. The $0.2=0.25\cdot 0.8$ value for car2, with 0.8 coming from the valuation _gew(10000,10500,5000) in evidence_price_le and 0.25 coming from 0.25 qPrice(10000,q2) (both are regarded as independent probabilistic events).

## Summary and Conclusion

We have presented in this paper various challenges and techniques to process PDatalog programs. As a description-oriented approach, PDatalog is an excellent technique to enable the formulation and separation of complex search strategies from the remaining code in a similar fashion like the separation from data and code drove the development of high-performing database technologies. Therefore the focus of this paper was on presenting the methods that were required to make PDatalog applicable in real-world scenarios with very large volumes of data.

The main contributions of this paper are as follows. Firstly, the paper reports some of the key developments in PDatalog in the last decade, in particular regarding scalability and efficiency, which are crucial to cope with the large volumes of data we are facing nowadays. Secondly, this paper reports for the first time insights of vague predicates and conditional atoms (the PDatalog level of the relational Bayes). Thirdly, the paper discusses extensive examples regarding the application and integration of factual and textual knowledge. This requires methods to describe the estimation of probabilities and methods to scale the probabilistic reasoning process to millions of tuples.

Section 2 has introduced and discussed selected features of the HySpirit system that are central to achieve an applicable PDatalog with respect to expressiveness, effectiveness and efficiency. In particular, we considered conditional atoms (the relational Bayes), vague predicates, probabilistic relational indexes, and parallel and distributed processing. Then, Section 3 focussed on applications. First, it demonstrated how to model text-based search. This was followed by the discussion of a more complex example combining factual and textual knowledge, vague predicates, an ontology, several retrieval strategies, and a functionality to explain the results.

The PDatalog implementation in HySpirit is capable of providing scalable and expressive means to create and process sophisticated retrieval strategies. The PDatalog layer in HySpirit itself is embedded in an abstraction hierarchy similar to programming languages. A probabilistic relational algebra (PRA) [16] is the ‘machine layer’ of the HySpirit stack on which PDatalog is built upon. PRA offers typical relational operations like SELECT, PROJECT, UNION, JOIN and SUBTRACTION in a probabilistic fashion. The HySpirit PRA operator BAYES estimates probabilities.

On top of PDatalog there are higher abstraction layers like the aforementioned four-valued probabilistic Datalog (FVPD) [8,10]. On top of these relational layers several object-relational abstraction layers are built which are tailored to a specific class of tasks, for instance structured multimedia document retrieval (POOL [6]) or retrieval incorporating (user) annotations (POLAR [3]). HySpirit is available on request by contacting the authors.

## Footnotes

1
https://lucene.apache.org/

2
http://www.lemurproject.org/indri/

3 There are automatic ways to compute the rule probability (using the Bayes operator), which would require a reformulation of this example that goes beyond the scope of this paper.

## References

[1] Azzam H, Yahyaei S, Bonzanini M, Roelleke T (2012) A schema-driven approach for knowledge-oriented retrieval and query formulation. In: Proceedings of the Third International Workshop on Keyword Search on Structured Data - KEYS '12. ACM, Scottsdale, AZ, USA. doi:10.1145/2254736.2254746. URL http://dl.acm.org/citation.cfm?doid=2254736.2254746

[2] Cornacchia R, Kamps J, Alink W, de Vries AP (2013) Searching political data by strategy. In: Lupu M, Salampasis M, Fuhr N, Hanbury A, Larsen B, Strindberg H (eds) Proceedings of the Integrating IR technologies for Professional Search Workshop. CEUR-WS.org, Moscow, pp 88–91. http://ceur-ws.org/Vol-968/irps_15.pdf

[3] Frommholz I, Fuhr N (2006) Probabilistic, object-oriented logics for annotation-based retrieval in digital libraries. In: Nelson M, Marshall C, Marchionini G (eds) Proc. of the 6th ACM/IEEE Joint Conference on Digital Libraries (JCDL 2006). ACM, New York, pp 55–64

[4] Fuhr N (2000) Probabilistic datalog: implementing logical information retrieval for advanced applications. J Am Soc Inf Sci 51:95–110

[5] Fuhr N (2014) Bridging information retrieval and databases. In: Ferro N (ed) Bridging between information retrieval and databases. Springer, Berlin, pp 97–115. doi:10.1007/978-3-642-54798-0fn{_}g5

[6] Fuhr N, Gövert N, Rölleke T (1998) DOLORES: a system for logic-based retrieval of multimedia objects. In: Croft WB, Moffat A, van Rijsbergen C, Wilkinson R, Zobel J (eds) Proceedings of the 21st Annual International ACM SIGIR Conference on Research and Development in Information Retrieval, pp 257–265. ACM, New York (1998)

[7] Fuhr N, Rölleke T (1997) A probabilistic relational algebra for the Integration of information retrieval and database systems. ACM Transactions on Information Systems 14, 32–66

[8] Fuhr N, Rölleke T (1998) HySpirit – a probabilistic inference engine for hypermedia retrieval in large databases. In: Proceedings of the 6th International Conference on Extending Database Technology (EDBT), pp 24–38. Springer, Heidelberg et al.

[9] Klampanos I, Azzam H, Roelleke T (2009) A case for probabilistic logic for scalable patent retrieval. In: CIKM Workshop on Patent Retrieval

[10] Lalmas M, Rölleke T (2003) Four-valued knowledge augmentation for structured document retrieval. Int J Uncertain Fuzziness Knowledge- Based Syst 11:67–85

[11] Ounis I, Amati G, Plachouras V, He B, Macdonald C, Lioma C (2006) Terrier: A High Performance and Scalable Information Retrieval Platform. In: Proceedings of ACM SIGIR'06 Workshop on Open Source Information Retrieval (OSIR 2006)

[12] Roelleke T (2003) A frequency-based and a Poisson-based probability of being informative. In: ACM SIGIR. Toronto, pp 227–234

[13] Roelleke T (2003) The relational Bayes for frequency-based and information-theoretic probability estimation in a probabilistic relational algebra. Patent application 0322328.6

[14] Roelleke T (2013) Information retrieval models: foundations and relationships. Morgan & Claypool. doi:10.2200/S00494ED1V01Y201304ICR027

[15] Roelleke T, Bonzanini M, Martinez-Alvarez M (2013) On the modelling of ranking algorithms in probabilistic datalog categories and subject descriptors. In: Proceedings of the 7th International Workshop on Ranking in Databases, 1, pp 4–9. Riva del Garda, Italy. doi:10.1145/2524828.2524832

[16] Roelleke T, Wu H, Wang J, Azzam H (2008) Modelling retrieval models in a probabilistic relational algebra with a new operator: the relational Bayes. The VLDB Journal - The International Journal on Very Large Data Bases, Special Issue on DB & IR 17(1):5–37. http://portal.acm.org/citation.cfm?id=1325167

